# The Integrated Tracking, Referral, and Electronic Decision Support, and Care Coordination (I-TREC) program: scalable strategies for the management of hypertension and diabetes within the government healthcare system of India

**DOI:** 10.1186/s12913-020-05851-w

**Published:** 2020-11-09

**Authors:** Shivani A. Patel, Hanspria Sharma, Sailesh Mohan, Mary Beth Weber, Devraj Jindal, Prashant Jarhyan, Priti Gupta, Rakshit Sharma, Mumtaj Ali, Mohammed K. Ali, K. M. Venkat Narayan, Dorairaj Prabhakaran, Yashdeep Gupta, Ambuj Roy, Nikhil Tandon

**Affiliations:** 1 Department of Global Health, Emory University, 1518 Clifton Rd NE / Rm 7037, Atlanta, USA; 2 All India Institute of Medical Sciences, New Delhi, India; 3 Centre for Chronic Disease Control, New Delhi, India; 4 Public Health Foundation of India, Gurgaon, India; 5 Department of Family and Preventive Medicine, Emory University, Atlanta, USA; 6 Department of Endocrinology & Metabolism, All India Institute of Medical Sciences, New Delhi, India; 7 Department of Cardiology, All India Institute of Medical Sciences, New Delhi, India

**Keywords:** Hypertension, Diabetes, Health system, Information technology, mHealth, Implementation science, Quality improvement, Continuum of care

## Abstract

**Background:**

Hypertension and diabetes are among the most common and deadly chronic conditions globally. In India, most adults with these conditions remain undiagnosed, untreated, or poorly treated and uncontrolled. Innovative and scalable approaches to deliver proven-effective strategies for medical and lifestyle management of these conditions are needed.

**Methods:**

The overall goal of this implementation science study is to evaluate the **I**ntegrated **T**racking, **R**eferral, **E**lectronic decision support, and **C**are coordination (I-TREC) program. I-TREC leverages information technology (IT) to manage hypertension and diabetes in adults aged ≥30 years across the hierarchy of Indian public healthcare facilities. The I-TREC program combines multiple evidence-based interventions: an electronic case record form (eCRF) to consolidate and track patient information and referrals across the publicly-funded healthcare system; an electronic clinical decision support system (CDSS) to assist clinicians to provide tailored guideline-based care to patients; a revised workflow to ensure coordinated care within and across facilities; and enhanced training for physicians and nurses regarding non-communicable disease (NCD) medical content and lifestyle management. The program will be implemented and evaluated in a predominantly rural district of Punjab, India. The evaluation will employ a quasi-experimental design with mixed methods data collection. Evaluation indicators assess changes in the continuum of care for hypertension and diabetes and are grounded in the Reach, Effectiveness, Adoption Implementation, and Maintenance (RE-AIM) framework. Data will be triangulated from multiple sources, including community surveys, health facility assessments, stakeholder interviews, and patient-level data from the I-TREC program’s electronic database.

**Discussion:**

I-TREC consolidates previously proven strategies for improved management of hypertension and diabetes at single-levels of the healthcare system into a scalable model for coordinated care delivery across all levels of the healthcare system hierarchy. Findings have the potential to inform best practices to ultimately deliver quality public-sector hypertension and diabetes care across India.

**Trial registration:**

The study is registered with Clinical Trials Registry of India (registration number CTRI/2020/01/022723). The study was registered prior to the launch of the intervention on 13 January 2020. The current version of protocol is version 2 dated 6 June 2018.

## Contributions to the literature

- The Government of India, beholden to its population of 1.3 billion, has developed an electronic “NCD Portal” that consists of an electronic case record form (eCRF) to manage non-communicable diseases (NCDs) within the government sector. The I-TREC program builds on the eCRF by integrating a proven-effective clinical decision support system for hypertension and diabetes care, accompanied by clinical training, to assist with patient management.
- We describe the evaluation protocol for the I-TREC multi-component strategy to improve diabetes and hypertensions care at all levels of the four-tier healthcare system in India.
- Lessons learned may inform optimal approaches to improve healthcare processes and health outcomes within the public sector healthcare system in India and in other similar settings.

## Background

Hypertension and diabetes together affect over 275 million Indians and their families [1]. These conditions are rising rapidly in all regions of India, commonly co-occur [2–4], and are associated with several adverse health outcomes—such as higher rates of death, myocardial infarction, stroke, blindness, kidney failure. Yet, both hypertension and diabetes are treatable such that timely and appropriate therapy mitigates associated morbidity due to complications and premature mortality. While lack of diagnosis is among the major obstacles to seeking appropriate treatment [5, 6], treatment outcomes even after diagnosis are far from ideal. Less than half of individuals who have hypertension and diabetes in the community are aware of their condition [7, 8], and only 20–25% achieve adequate blood pressure [9] or blood glucose control [10]. Under-diagnosis, under-treatment, and poor control for both hypertension and diabetes are disproportionately high in rural settings [11, 12], where the majority of the Indian population resides.

India’s rural healthcare system is currently organized as a hierarchy of facilities that range from relatively lower-skilled personnel supported by simple infrastructure at the village level to relatively higher-skilled personnel supported by sophisticated infrastructure at the district level. This model attempts to maximize geographical coverage by allowing for “up referrals” and “down referrals” across levels of the healthcare system so that the demand of the individual patient can be met by appropriate resources, such as skilled human resources, infrastructure and services. The referral linkages between these institutions, while theoretically in place, are not implemented efficiently or cohesively. In practice, patients access any level of the healthcare system convenient for them, resulting in a mismatch between patient needs and resource availability. Challenges to the system are compounded by the heterogeneity of treatment guidelines, diagnostic modalities, and medications [13–17] needed at all levels of health care to appropriately serve the growing population with NCDs alongside the large population seeking care for maternal and child health and infectious diseases [18–21].

Recognizing the growing burden of hypertension and diabetes across all segments of the population, the Ministry of Health & Family Welfare, Government of India, has taken the initiative to integrate screening and management of these conditions into primary care under its National Health Mission (NHM) and the National Programme for Prevention and Control of Cancer, Diabetes, Cardiovascular Disease, and Stroke (NPCDCS). A major component of the government strategy is to encourage universal screening for hypertension and diabetes of adults aged ≥30 years in the community and subsequent referral of potential cases to higher level facilities. Consequently, the expected volume of adults seeking care for hypertension and diabetes at government health facilities is anticipated to surge. Building upon the tremendous need and political will to identify optimal and scalable approaches to expand successful care models to manage blood pressure and diabetes within the public healthcare system, we developed the **I**ntegrated **T**racking, **R**eferral, **E**lectronic decision support, and **C**are coordination (I-TREC) program. We describe the components of the I-TREC program and its evaluation design.

## Methods/discussion

### Setting and target population

I-TREC was developed as a collaboration between the All India Institute of Medical Sciences, New Delhi (AIIMS), the Centre for Chronic Disease Control (CCDC), and Emory University. For over a decade, these three institutions have collaboratively developed and tested the combination of information technology (IT), enhanced personnel training, and workflow alterations to improve the quality of care for diabetes and hypertension in diverse settings across India [22–29]. Most of these prior efforts focused on a single level of the healthcare system and relied on research staff to implement the intervention. In I-TREC, however, our goal was to develop and evaluate a coordinated package of tested tools and provider training approaches that catered to functions and personnel available in each type of the 4-tier healthcare system (see Table 1). We further sought to embed the program within the infrastructural scaffolding provided by the Government of India in the interest of future scalability. I-TREC was thus designed to be implemented by personnel and using resources (medications, diagnostics) already present within the public healthcare system.

**Table 1:** Intervention components

Healthcare facility level	I-TREC components	Available Staff	Tasks and functions
**Level 1: Village Sub-Centre**	eCRF	ANM	Universal screening of hypertension and diabetes for adults ages ≥ 30 y in the community Enrol community members into the NCD portal Adults with blood pressure ≥ 140/90 or random blood glucose ≥140 mg/dl referred to medical officer at nearest facility (level 2) for confirmation and initiation of treatment
**Level 2: Primary Health Centre**	eCRF+CDSS	SN, MO	Confirmatory diagnosis of hypertension and diabetes of suspected cases referred from sub-centre Routine management of adults with stable hypertension and diabetes Generate and update eCRF Use CDSS to develop treatment plan and determine need for up-referral
**Level 3: Community Health Centre**	eCRF+CDSS	SN, MO	Run dedicated NCD clinics Routine management of adults with stable hypertension and diabetes Generate and update eCRF Use CDSS to develop treatment plan and determine need for up- or down-referral
**Level 4: District Hospital**	eCRF+CDSS	SN, MO	Secondary care available for all health conditions and complications Management of medically complex patients with hypertension and diabetes Generate and update eCRF Use CDSS to develop treatment plan and determine need for up- or down-referral

*ANM* Auxiliary nurse midwife, *CDSS* Clinical decision support system, *eCRF* Electronic case record form, *MO* Medical officer (physician), *SN* Staff nurse

**Fig. 1:**
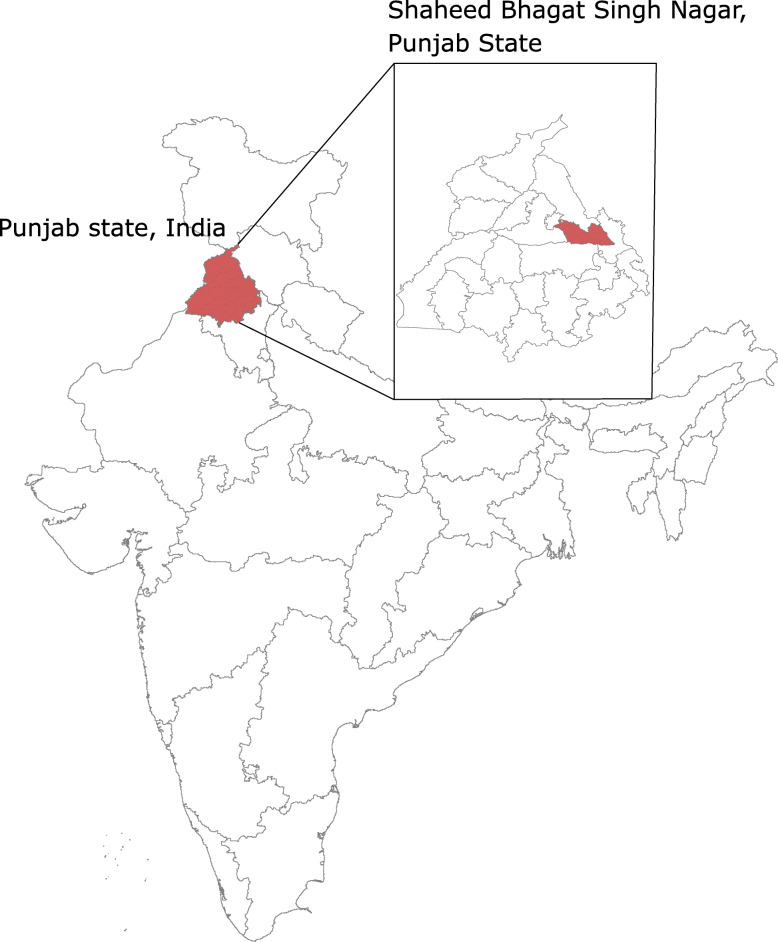
Geographical location of the study setting. Figure 1 was created by the authors using a map taken from Map Data© 2020 Google

The primary implementation partners for the program include the Department of Health and Family Welfare, Government of Punjab (use of IT tools and altered work flow to deliver routine care); TATA Trusts (conduct training of healthcare workers and technical assistance); and Dell Technologies (development of software and IT infrastructure). Monitoring and evaluation activities for I-TREC will be conducted by AIIMS, CCDC, and Emory University. I-TREC will be implemented in Mukandpur block of Shaheed Bhagat Singh Nagar district, Punjab, India, and evaluated through comparison of program indictors with those observed in the neighboring Sujjon block in the same district (See Fig. 1). The program and comparison locations were selected based on consultation with the Punjab Department of Health and Family Welfare.

### Program components

Many barriers to optimal hypertension and diabetes care can be alleviated through IT-based quality improvement strategies. Clinical decision support software can provide up-to-date guidance to clinicians [30] to manage hypertension and diabetes following standard treatment protocols. Electronic health records can ensure that access to historical patient data and course of illnesses are available to clinicians to guide clinical decisions at whichever facility the patient enters. IT tools can also help make referral linkages between the different levels of healthcare more transparent, efficient, and effective by suggesting referral thresholds to clinicians, notifying facilities of referred patients, and maintaining a record of recommendations to refer the patient to. Finally, digitized systems to track and monitor case management can incentivize improved health provider performance.

Motivated by the potential benefits IT tools offer clinicians and health systems, the I-TREC program includes: an electronic case record form (eCRF) to consolidate and track patient information; an electronic clinical decision support system (CDSS) for clinicians to provide tailored guideline-based care to patients with in-built prompts for triggering referrals across health facilities as needed; a revised workflow to ensure coordinated care within and across facilities; and enhanced training for clinicians regarding NCD medical content and lifestyle management (See Table 1). Each component of this integrated system is described below.

#### Electronic case record form (eCRF)

The eCRF is the Government of India’s digitized health record focused on compiling patient data relevant to chronic diseases. The eCRF allows nurses to enter patient demographic information, medical history, physical examinations, and laboratory investigations into an electronic form through a web-based “NCD portal.” Patient data are then stored in a cloud server hosted by National Informatics Centre, Government of India. The eCRF ensures that necessary patient medical history will be available seamlessly up and down the healthcare facility hierarchy, reduces redundant data entry when the same patient seeks care at different facilities, and allows clinicians to track patient health information over time across visits. Simultaneously, these data serve as inputs for the CDSS to provide guideline-based recommendations to clinicians to optimize medical and lifestyle management and referral of patients. The eCRF itself was developed by a committee of experts across India, including members of the I-TREC investigator team (NT and AR). We chose to build upon the Government of India’s eCRF to align our program with the national effort to incorporate IT into the management of NCDs in the public sector.

#### Clinical decision support system (CDSS)

The CDSS generates customized evidence-based treatment advisories for patients with hypertension and diabetes. The treatment advisories are based on up-to-date national and international guidelines that were further vetted by our expert clinical investigators, and tailored to each level of health facility (primary, secondary or tertiary), for example, by taking into consideration the local availability of medications and diagnostic capability. The CDSS algorithms provide the clinician with an instantaneous advisory regarding medication titration based on patient history and current clinical examination as inputted into the eCRF. The attending clinician has the option of rejecting, partially accepting, or fully accepting the advisory to generate a final treatment plan. In addition to the treatment plan, the CDSS has in-built prompts to refer patients up or down the healthcare facility hierarchy to direct patients to the most appropriate level of care for ongoing disease management. The treatment plan, referral instructions, and lifestyle advice specific to the patient become a part of the patient’s eCRF and are also printed out on paper and given to the patient.

#### Healthcare provider training

Healthcare providers employed in both the program and comparison blocks receive refresher content training related to the etiology, behavioral counseling, and medical management of NCDs following established NPCDCS training manuals. The content training is provided over a full day in separate sessions for auxiliary nurse midwives [ANMs], staff nurses, and medical officers. The sessions include training on effective techniques for delivering behavioral and lifestyle advice counselling. Unlike routine training, this refresher training includes innovative learning methods, such as case studies and role playing to enhance trainee engagement to assure improved comprehension and retention of behavioral and lifestyle counselling approaches. In addition, staff nurses and medical officers in the program block receive IT training on the use of the eCRF and CDSS, specific to their level of expertise and the level of healthcare facility in which they are employed.

#### Patient flow under I-TREC

**Fig. 2:**
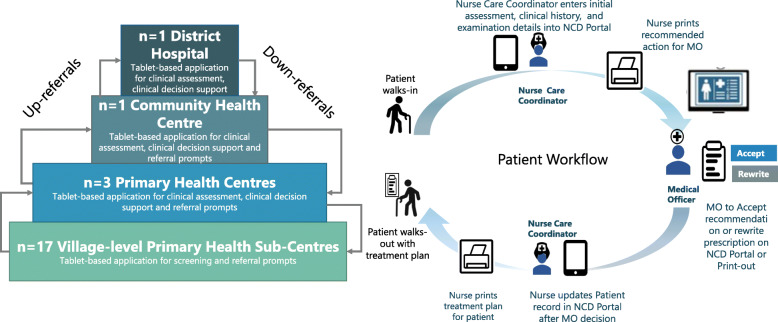
Patient flow under the I-TREC program. Panel **a** shows patient flow across facilities and Panel **b** shows patient flow within facilities. Figure 2 was developed by the authors

Figure 2 depicts patient flow within and across facilities in the I-TREC program. Following NHM and NPCDCS recommendations, all adults aged ≥30 years are eligible for universal screening of hypertension and diabetes in the community and opportunistic screening in health facilities by government health providers. At the village-level Sub-Centre, the lowest level of the healthcare facility hierarchy, the ANM is tasked with screening adults to identify suspected cases of hypertension and diabetes in the community. ANMs enter screening results into the “ANM portal” using a tablet-based application. Adults who are suspected to have hypertension and/or diabetes are referred to the nearest Primary Health Centre, the second level of the healthcare hierarchy, for diagnosis and treatment (see Table 1 and Fig. 2).

At Primary Health Centres and above—namely Community Health Centres and the District Hospital—nurses generate and update the patient eCRF through a web-based application on a computer tablet. At the time the eCRF is first generated, nurses record the patient’s clinical history. At future visits, the eCRF is updated with ongoing examination data so that the CDSS is responsive to the patient’s health status at a given visit. Patient data entered into the eCRF are uploaded to a secure cloud-based server once per day, and these data are synced and retrievable at all facilities to inform the CDSS and assist with clinical decisions.

After the initial eCRF review and update by the nurse, patients with confirmed hypertension or diabetes are instructed to see the medical officer, who is aided by the CDSS to manage these conditions. The CDSS algorithms are tailored to the expertise, medications, and diagnostic tests available at each level of facility and customized to the clinical history of the patient over all past and present contacts with the health system. Medically complex patients, such as those who are resistant to therapy, may be referred further “up” the referral hierarchy. The referral algorithms take into account a patient’s full clinical history and current health profile, including the number of medications currently prescribed, treatment response based on laboratory investigations, and comorbidities. For example, if a patient with diabetes under treatment at a PHC has uncontrolled hyperglycemia despite being on the maximum tolerated dose of three oral hypoglycaemic agents, the CDSS will trigger a referral to a CHC. Similarly, if a patient with hypertension under treatment at a CHC level has uncontrolled blood pressure despite being on the maximum tolerated dose of two antihypertensive drugs, the CDSS will trigger a referral to the District Hospital.

Once the patient achieves a stable clinical state, she or he will be referred back “down” to the lowest level of health facility (PHC or CHC) that is suitable for routine management of stable disease and dispensation of appropriate medication.

#### Routine care

Adults residing in the comparator block will continue to receive the usual care by local physicians and nurses using paper-based record systems and without the assistance of the CDSS.

### Evaluation

#### Design

We will employ a two-group pre-post quasi-experimental design to conduct a mixed methods evaluation of the I-TREC program in Punjab, India. While the I-TREC program is implemented within the health system by clinicians, we will assess indicators at the levels of facilities, clinicians, patients, and communities.

#### Ethics

The evaluation protocol was reviewed and approved by the ethics committee at All India Institute of Medical Sciences (AIIMS), New Delhi, India (IEC-361/07.07.2017). Given that the Government of Punjab will be implementing I-TREC, and role of research partners is limited to program design, training, and evaluation, this study was deemed to be observational. The role of researchers at Emory University, Atlanta was deemed Not Human Subjects Research (IRB00098808). Participants from whom our research team collects data for evaluation purposes will provide written informed consent following procedures approved by the AIIMS Ethics Committee.

#### Outcomes

Program indicators are guided by the Reach, Effectiveness, Adoption Implementation, and Maintenance (RE-AIM) framework [31]. The principal endpoints for evaluation are shown in Table 2, and focus on the domains of reach, effectiveness, adoption, and implementation of the program components. Reach and effectiveness will be assessed using a combination of community-based data and facility-based data. For example, the proportion of adults ages 30 and older in the community who are screened for hypertension is a measure of reach that will be obtained through a representative community survey. A second measure of reach is the number of patients seeking care for hypertension and diabetes who have an eCRF, which will be measured through health facility data. Similarly, effectiveness will be assessed through health outcomes (e.g., reductions in mean blood pressure and/or mean blood glucose) among patients attending program facilities and also among adults with hypertension and/or diabetes in the community. Adoption metrics focus on healthcare provider utilization of the eCRF and CDSS tools. Implementation measures focus on quantifying the proportion of patients who receive care through the eCRF and CDSS tools. Finally, maintenance will be assessed through qualitative research with stakeholders within the health system to understand views of sustainability.

**Table 2:** Key outcome indicators for the I-TREC evaluation

RE-AIM domain	Key indicators
**Reach**	Proportion of adults ages 30 and older in the community screened for hypertension by a government healthcare provider
	Proportion of adults ages 30 and older in the community screened for diabetes by a government healthcare provider
	Number of patients seeking care for hypertension and diabetes at a government health facility who have an eCRF
**Effectiveness**	Reduction in mean blood pressure in patients receiving care in program facilities
	Reduction in mean blood glucose in patients receiving care in program facilities
	Proportion who achieve blood pressure and blood glucose control among patients receiving care in program facilities
	Proportion who achieve blood glucose control among patients receiving care in program facilities
	Reduction in mean blood pressure in the community
	Reduction in mean blood glucose in the community
	Proportion of hypertension patients who achieve blood pressure control in the community
	Proportion of diabetes patients who achieve blood glucose control in the community
**Adoption**	Proportion of healthcare providers (by type) who log into the NCD portal
	Proportion of clinicians who fully or partially accept CDSS prompts
	Proportion of healthcare providers (by type) who report satisfaction with the eCRF+CDSS
**Implementation**	Percentage of hypertension patients who received guideline-based care through the eCRF+CDSS (of all registered patients with hypertension)
	Percentage of diabetes patients who received guideline-based care through the eCRF+CDSS (of all registered patients with diabetes)
	Percentage of hypertension patients who made repeat visits to health facility
	Percentage of diabetes patients who made repeat visits to health facility
	Percentage of “up-referral” cases who attend appointments
	Percentage of patients who were seen at a higher level facility that returned to the Sub-Centre for ongoing management (“closing the referral loop” and ensuring continuity of care)
	Percentage of patients tracked with multiple visits over the course of the program
	Mean time for data upload from each level of facility to central server
**Maintenance**	Views of program sustainability and barriers to sustaining and disseminating the program (qualitative)

#### Sources of data for evaluation

Data will be triangulated from multiple sources, including facility and patient assessments, stakeholder interviews, community surveys, and patient-level data from the I-TREC electronic database. Where appropriate, data will be collected prior to the program launch and again following 36 months of the program. With the exception of the I-TREC eCRF-CDSS data, evaluation data will be collected by trained research staff.

#### Health facility and patient assessments

Pre- and post-program health facility and patient assessments include 1) a health facility form; 2) patient flow mapping; and 3) patient out-of-pocket cost of care surveys. Health facility forms will be completed at all 52 government health facilities (21 in program and 30 in control and the common District Hospital) to describe the infrastructure, facility personnel and salaries, availability of medications, and availability of diagnostics and laboratory investigations. Health facility form completion requires a combination of observational checklists and structured interviews with administrators. Patient flow mapping entails identifying and following patients with hypertension and diabetes through typical visits to map the typical workflow, diagnostic and prescription practices, and duration of visits. Together, the health facility form and patient flow mapping will provide data to describe the resources (time and costs) associated with typical healthcare visits for patients with hypertension and diabetes with and without the I-TREC program. Patient cost surveys will be used to obtain data on expenditures related to outpatient and inpatient health care utilization in the last 3 months to understand the cost incurred by patients to manage their disease. Administration of the pre- and post-program patient cost surveys will contribute data to understand whether the program has any impact on patient expenditures related to hypertension and diabetes. Purposive sampling will be done to recruit patients for the patient flow mapping and cost surveys.

#### Stakeholder perspectives

Qualitative methods will include a combination of focus group discussions of the community members, key informant interviews with healthcare providers and in-depth interviews of patients to provide a richer interpretation of quantitative findings and explore the processes underlying the uptake and delivery of the I-TREC program. The qualitative research will be conducted before, during, and after program implementation. Purposive sampling will be done to recruit information-rich participants for interviews and focus group discussions. For all qualitative data analysis, the textual data (verbatim transcripts created from digital recordings of interviews and focus group discussions) will be reviewed to identify key themes and domains of interest. A code book will then be developed to reflect these domains and include both inductive (derived from the textual data) and deductive (based on literature and theory) codes. Inter-coder reliability will be assessed, and the codebook will be finalized and applied to the data. The codebook will include codes specific to each type of data collection and shared codes across participant type. A thematic analysis will be used to describe individual- or community-level views on discussion topics including program barriers and facilitators, community barriers, views of the healthcare system, and acceptability and feasibility of the program.

#### Pre- and post-program quantitative cross-sectional community surveys

The community-based evaluation component will assess whether the I-TREC program has an impact on blood pressure and blood glucose awareness, treatment seeking, and control among adults in the community. This evaluation component is critical to learning the real-world impact of the I-TREC program on community-level indicators of the care continuum (screening, treatment, control). Data collected in the program and comparison blocks prior to the program will be compared with data collected from these same blocks after the program using identical procedures. This design allows us to assess and address several threats to validity, including lack of temporal order, comparability across the two blocks (leading to potential confounding by population composition) and secular changes unrelated to our program that affect study endpoints (leading to potential confounding by external factors). Given that we will be sampling separate cross-sections of the population in each group and time point, we do not expect inference to be affected by population aging (maturation threats) over the 3-year program period.

At baseline—prior to intervention—we employed a multi-stage cluster sampling design to obtain a representative sample of adults aged 30 years and older in both blocks under study. Within each block, census data were used to select villages proportionate to population size and subsequently we conducted household mapping and listing to generate a sampling frame for households. Households were selected using systematic random sampling, and one adult man and woman from each household were randomly selected using the Kish method to achieve the desired sample size. At endline, this same procedure will be repeated.

The community survey sample size was determined to estimate differences in mean reduction in systolic blood pressure among those with diagnosed hypertension in the community. First, we computed the base sample size required to detect a desired effect size of 5 mmHg given the SBP standard deviation of 18.5, power = .80 and α = .05, based on the mPower Heart Study [24]. We estimated that 168 individuals with hypertension would be needed to detect the anticipated effect size. Second, we estimated that we would require a sample size of 839 adults in the general population to identify 168 individuals with hypertension, assuming prevalence of diagnosed hypertension of 20%. Third, we determined the optimal sample allocation for a multi-stage sampling design that would be time-efficient for field work and statistical precision. We assumed an intraclass correlation of systolic blood pressure of 0.018 based on village-level clustering of SBP in the DISHA study [32] (unpublished findings). After applying a 10% refusal rate based on our prior field studies in the region, we determined that a cluster size of 50 adults per village distributed across 35 villages per block was optimal. This yielded a total sample size of 3508 for the community survey to be evenly split between the program and comparison blocks.

#### Health outcomes among patients receiving the I-TREC program

Using patient health data from the eCRF, we will assess processes of care and changes in blood pressure and blood glucose outcomes over time among patients with hypertension or diabetes who seek care at I-TREC program facilities in a facility-based evaluation component. Because I-TREC is being integrated into the routine care in the program block under real-world conditions, we will not be assigning individual patients to treatment nor actively following patients for research visits. Rather, all adults residing in the I-TREC catchment area (i.e., residents of Mukandpur block) will be exposed to the program and patient data will be collected every time a person chooses to receive care at a government health facility. Data from all patients visiting facilities in the program block captured in the Government of India eCRF will be de-identified and obtained by AIIMS throughout the program period for monitoring and evaluation purposes.

The sample size for the facility-based evaluation is out of our control and contingent on the number of patients who seek care at government facilities. We therefore report the detectable effect size for longitudinal change in systolic blood pressure over time in patients at I-TREC facilities after setting power to 80% and α = .05. The program block, Mukandpur, has a population of 98,000. We expect that 30% of the local population will seek care at a government facility, 50% will be age-eligible (30 years and older) per the government guidelines for universal and opportunistic screening of hypertension and diabetes, and 20% will test positive for hypertension, amounting to an estimated patient pool of 2940 adults. Assuming that 50% of all enrolled patients with hypertension and diabetes make repeat visits (enabling us examine changes in outcomes), we will be able to detect a 1.35 mmHg difference in SBP.

#### Process measures

In the program block, we will examine measures of adoption and implementation of the IT tools, such as completeness of eCRF forms, acceptance (partial and full) and rejection of the CDSS advisories, time stamp of data entry, the initials of the enterer, and average number of new records per day. In both the program and comparison block, data regarding the total number of patients recorded in the out-patient registry at the facility, numbers screened for hypertension and diabetes, numbers receiving medication from the pharmacy, and numbers referred to higher level facilities will be collected through a combination of paper-based registries and routine NPCDCS reports. The I-TREC evaluation team will obtain these facility-level data using abstraction forms without removing any paper records from premises. In addition, the I-TREC evaluation team will periodically conduct random, unannounced visits to directly observe the number of patients seeking care for hypertension and diabetes facilities in both blocks. Additional data on intervention fidelity measures (e.g., use of eCRF during health visit, measurement of blood pressure and blood glucose, provision of the I-TREC print out to the patient) will also be collected through patient exit interviews and the eCRF backend data.

#### Statistical analysis plan

Quantitative data analysis will be performed using SAS, STATA, and R software. Descriptive analyses of the community-based data will examine socio-demographic characteristics, health indicators, and healthcare behaviors of the program and comparison block samples at baseline and end-line. The quantitative evaluation of health and healthcare endpoints will focus on assessing changes in the continuum of care indicators and mean blood pressure in the community-based surveys. We will assess changes in baseline to end-line indicators of health outcomes (e.g., blood pressure) and changes in continuum of care indicators (e.g., proportion screened) for both the program and comparison blocks; see Table 2 for indicators. A simple difference-in-difference (DiD) [33] estimate for each indicator will be computed as

$$ \mathrm{DiD}=\left({p}_{g=\mathrm{i},\mathrm{t}=1}-{p}_{g=\mathrm{i},\mathrm{t}=0}\right)-\left({p}_{g=\mathrm{c},\mathrm{t}=1}-{p}_{g=\mathrm{c},\mathrm{t}=0}\right) $$

where p indicates prevalence or mean of each indicator; *g* subscripts group (i = program; c = comparison); and t subscripts the time point of data (0 = pre-program; 1 = post-program). We will estimate log-binomial models (binary outcomes) or linear models (continuous outcomes, with log-transformation if needed) with robust variance to compute the DiD after accounting for compositional characteristics of the community and clustering of data within villages. For each outcome indicator separately, the following model will be estimated using individual-level data:

$$ \mathrm{Outcome}\ \mathrm{indicator}\sim \mathrm{program}\ \mathrm{group}+\mathrm{pre}-\mathrm{post}\ \mathrm{indicator}+\mathrm{program}\ \mathrm{group}\ \mathrm{x}\ \mathrm{pre}-\mathrm{post}\ \mathrm{indicator}+\mathrm{age}+\mathrm{sex}+\mathrm{education}+\mathrm{religion}+\mathrm{marital}\ \mathrm{status}+\mathrm{below}\ \mathrm{poverty}\ \mathrm{line}+\mathrm{facility}\ \mathrm{type}\ \left(\mathrm{public}\ \mathrm{versus}\ \mathrm{private}\right). $$

The coefficient associated with the interaction term, “program group x pre-post indicator,” is the adjusted DiD estimate accounting for heterogeneity in socio-demographic characteristics. The model will be estimated using generalized estimating equations (GEE) to account for clustering of outcomes within the villages (i.e. village is the cluster variable specified for statistical analysis). Sub-group analyses will examine differences by gender and socioeconomic status.

Data points recorded in the I-TREC system will be analyzed by month to examine trends over time and seasonality. We will also evaluate change patient outcomes over time (e.g., mean SBP change). While several of the I-TREC platform indicators are purely descriptive measures of performance (e.g., mean time for data upload), other indicators of healthcare delivery may be compared between I-TREC facilities and comparison group facilities (e.g., patient volume).

### Summary

This is the first study in India evaluating the composite impact of a clinical decision support system integrated with the Government of India eCRF, combined with modified patient flow and enhanced healthcare provider training. It is also an important early effort to systematically evaluate a program for integrated management of hypertension and diabetes at all levels of the public healthcare system, starting from the sub-centre up to the district hospital. Lessons learned may inform optimal approaches to improve healthcare processes and health outcomes within the public sector healthcare system in India.

The I-TREC program and its evaluation have several strengths but also some limitations. Given the primacy of scalability, the role of research staff is limited to program design, training, monitoring and evaluation. Therefore, the context and conditions of implementation are beyond the control of investigators. For example, the availability of drugs, diagnostic investigations, and clinicians are likely to impact the reach, effectiveness, and implementation of I-TREC but rest in the hands of the state government. Nevertheless, we expect variations in these structural elements to affect both the program and comparison blocks similarly. In order to maintain comparability between the program and comparison block, neighboring blocks within the same district were chosen. However, this means that both blocks share the district hospital, which will have to be taken into account during the analysis. While we will be able to obtain patient data in the program block from the eCRF, no comparable source of data is available to use in the comparison block. Moreover, undue monitoring of the comparison block may inadvertently lead to compensation behaviors on the part of clinicians that could undermine our ability to measure performance differences across the two blocks. The community survey, in part, is designed to mitigate these limitations by providing a well-designed comparison of healthcare processes and outcomes as observed in the program and comparison blocks.

This implementation research is intended to provide evidence of workable programs to manage chronic diseases in India and inform the evolving NPCDCS [34]. Given the similarities in the health system and epidemiologic transition between in India and other low- and middle-income countries, this research has additional scope to potentially inform best practices for management of hypertension and diabetes outside of India. In fact, members of our team (DJ, AV, DP) have collaborated with the World Health Organization (WHO)-Southeast Asia Regional Office and the Republic of Maldives for the development of the “mPEN App,” which is a CDSS tool that draws on similar technologies as what is described here to implement the WHO Prevention of Essential NCDs package in primary health care in the Maldives. We envision that the I-TREC program and evaluation will provide opportunities for continued cross-national collaborations and idea exchange to improve hypertension and diabetes care globally.

### Current status

Intervention development and pre-testing were completed in August 2019. Healthcare providers in both blocks received training in December 2019, and the I-TREC program was launched in January 2020. Prior to the program launch, pre-program data collection, including facility and patient assessments, qualitative research, and community survey, were completed. Since March 2020, both intervention roll-out and research activities have been impacted by the COVID-19 pandemic. Specifically, government healthcare system resources have been diverted from NCD care to test and treat patients with SARS-COV-2, national and local lockdown measures have forced intermittent closures of lower-tier health facilities and prevented field staff from conducting routine monitoring activities. In addition, patient flow through facilities—when open—has generally declined, possibly due to fear of contracting the virus while seeking healthcare. Nevertheless, as and when healthcare facilities are operational, the intervention components are being implemented by nurses and physicians in the program facilities and program monitoring activities are underway. We expect intermittent disruptions to intervention implementation and monitoring activities to continue until the COVID-19 pandemic has been fully controlled.

## Data Availability

Data generated from this study will be made available to researchers upon request. Requests may be made to Dr. Patel and will be processed through the I-TREC Data Sharing and Publications Committee.

## Abbreviations

ANM: Auxiliary nurse midwives
AIIMS, CCDC: All India Institute of Medical Sciences, New Delhi (AIIMS), the Centre for Chronic Disease Control
CDSS: Clinical decision support system
DiD: Difference-in-difference
eCRF: Electronic case record form
IT: Information technology
I-TREC: Integrated Tracking, Referral, and Electronic Decision Support, and Care Coordination Program
NCD: Non-communicable disease
NHM: National Health Mission
NPCDCS: National Programme for Prevention and Control of Cancer, Diabetes, Cardiovascular Disease, and Stroke
RE-AIM: Reach, Effectiveness, Adoption Implementation, and Maintenance
